# Empathy, Burnout, and Attitudes towards Mental Illness among Spanish Mental Health Nurses

**DOI:** 10.3390/ijerph19020692

**Published:** 2022-01-08

**Authors:** Daniel Román-Sánchez, Juan Carlos Paramio-Cuevas, Olga Paloma-Castro, José Luis Palazón-Fernández, Isabel Lepiani-Díaz, José Manuel de la Fuente Rodríguez, María Reyes López-Millán

**Affiliations:** 1 Nursing Faculty “Salus Infirmorum”, University of Cadiz, 11001 Cadiz, Spain; daniel.roman@ca.uca.es (D.R.-S.); paramio@msn.com (J.C.P.-C.); joseluispalazonf@gmail.com (J.L.P.-F.); isabel.lepiani@gmail.com (I.L.-D.); jfuenter@hotmail.com (J.M.d.l.F.R.); 2 Nursing Department, University of Cadiz, 11001 Cadiz, Spain; olga.paloma@uca.es

**Keywords:** acute mental health, empathy, nursing role, stigma

## Abstract

Mental health nurses, together with psychiatrists, are the healthcare professionals who display the highest levels of empathy and the best attitudes towards patients with mental disorders. However, burnout is a common problem among these professionals. The aim of our study is to describe the association between empathy, burnout, and attitudes towards patients with mental disorders among mental health nurses in Spain. A descriptive cross-sectional design was used involving a sample of 750 specialist nurses working in mental health facilities in Spain. An intentional, non-probability, non-discriminative, exponential snowball sampling method was used. The Jefferson Scale of Empathy, the Maslach Burnout Inventory, and the Community Attitudes towards Mental Illness Inventory were used to measure the study variables. A positive correlation was observed between empathy and all the study variables, with the exception of the personal accomplishment dimension of burnout and the social restrictiveness and authoritarianism dimensions of attitudes towards mental illness, where a negative relation was observed. Our findings suggest that empathy is associated with an increase in positive attitudes towards patients with mental disorders, decreasing associated stigma, but did not act as a protective factor against burnout in the study sample.

## 1. Introduction

The stigma of mental illness is a reality in our society, especially among healthcare professionals [1,2]. Empathy among mental health nurses is a key element helping to reduce stigma towards patients with mental disorders [3]. In addition, mental health nurses often experience the well-known burnout syndrome, which reduces the quality of their professional work [4]. An analysis of the associations between these variables may provide a better understanding of how they interact among mental health nurses working directly with patients with mental disorders in Spain.

Empathy is a multidimensional construct [5] responsible for perceiving the experiences, concerns, and perspectives of another individual without losing reference to one’s own self [6]. Empathy may be modified by receiving training and through individual and work experience [7].

Multiple benefits related to empathy have been described in the healthcare setting, such as improved healthcare, improved professional-patient relations, better adherence to treatment, better clinical outcomes, and increased patient satisfaction, and increased satisfaction in healthcare professionals [8,9,10]. Moreover, empathy is the interpersonal skill most highly valued by both healthcare professionals and patients [11]. However, the international literature reports that there are significant differences between men and women [12,13,14].

It has also been shown that the number of years worked, stressful experiences, lack of time, lack of contact with other professionals, and pressure on the health system make it difficult for healthcare professionals to interact empathetically, thereby empathy decreases and burnout increases [15]. This syndrome has been reported to be experienced by between 30% and 40% of nurses worldwide [16] and is described as a set of symptoms including depersonalization, emotional exhaustion, and lack of personal accomplishment [17], leading to communication problems and a poor understanding of patients’ needs [16]. In these cases, there is evidence that high levels of empathy act as a protective factor against burnout [18].

Nursing is the health profession with the highest levels of empathy in its relation with patients according to Petrucci et al. [19]. The construct of empathy is considered a key communication skill for nursing [20] and therefore, a number of studies on empathy have focused on nursing professionals [21].

Within the field of nursing, the mental health nursing specialty bases most of its actions on communication therapies [22], in which empathy helps to reduce negative attitudes towards patients with mental disorders. Tippin and Maranzan [3] argue that improving attitudes towards mentally ill patients decreases stigma, which is considered a negative emotional, behavioral, or cognitive response to individuals with a mental disorder [23,24,25]. A recent review of the literature reaffirms that empathy continues to be an essential component of the nurse–patient therapeutic relation in current mental health units [26]. For this reason, mental health nurses are the health professionals who, together with psychiatrists, display the highest levels of empathy and the best attitudes towards users with mental disorders [27,28].

A number of studies suggest that empathy is one of the factors that, together with the experience of caring for mental health patients, protects mental health nurses against burnout and increases the bond with mentally ill patients by positively modifying their attitudes towards them [29].

Empathy, burnout, and the relation between them among mental health nurses have been studied previously. Often independently or relating only to empathy and burnout [12,30]. However, our study is the first to address the relation between empathy, burnout, and attitudes towards patients with mental disorders among mental health nurses in the Spanish national health system. Moreover, few studies have been conducted on the levels of empathy among mental health nurses [12,31]. We can add that the number of studies that investigate these variables is small and most of the studies have not been carried out in Spain [3,12,28,31,32].

We believe that it would be desirable for mental health nurses to have high levels of empathy and attitudes towards patients with mental disorders, as well as low levels of burnout.

The aim of this study is thus to describe the levels of empathy, burnout, and attitudes towards patients with mental disorders among mental health nurses and their sociodemographic variables and to describe the relation between these variables. We hypothesized that high empathy (independent variable) is related to lower burnout (lower emotional exhaustion, lower depersonalization and higher personal accomplishment) and better attitudes towards patients with mental disorders (lower authoritarianism and social restrictiveness, higher benevolence and community mental health ideology).

## 2. Materials and Methods

### 2.1. Study Design and Participants

This is a descriptive cross-sectional study conducted at mental health facilities in the Spanish national health system. This research adheres to the STROBE guidelines [33] for the reporting of observational studies. All nurses who met the eligibility criteria were included in the study: (i) being a mental health nurse specialist, and; (ii) performing their healthcare work in mental health facilities in the Spanish national health system at the time of the study. An intentional, non-probability, non-discriminative, exponential snowball sampling method was used [34] aiming to recruit eligible subjects from different parts of Spain. This type of sampling helps us to access hard-to-reach populations, expand the sample size and facilitate data validation. Sampling starts from the selection of the initial collaborators called “seeds”, these will not enter the subsequent analysis. The seeds select a certain number of new participants that will be useful for the analysis and so on [35]. This study involved an initial population of 4000 nurses working in mental health facilities in the Spanish national health system, as estimated by the Spanish National Association of Mental Health Nurses (Asociación Nacional de Enfermería de Salud Mental) [36]. To recruit the study subjects, we had collaborators in the different mental health units of the different Spanish autonomous communities (seeds). The measurement instruments necessary for carrying out the study were disseminated through them, making them available to the maximum number of mental health nurses possible. For the recovery of the data, personal contact was made with the collaborators of the units where the procedure needed to be carried out. All mental health facilities were contacted by email and 750 nurses agreed to participate in the study by completing an anonymous online questionnaire on empathy, burnout, and attitudes towards mentally ill patients between March and November 2019. Participants gave informed consent and their data were coded to ensure confidentiality.

### 2.2. Instruments and Variables

- Empathy. Empathy was measured using the Spanish version of the Jefferson Scale of Empathy (JSE) [37] containing 20 items related to empathic response in patient care settings. Ten of them are worded positively and the other 10 are worded negatively to avoid unreflective answers. Each item is scored on a 7-point Likert scale ranging from 1 (strongly disagree) to 7 (strongly agree). Scores are reversed for negative items, which means that individuals responding ‘strongly disagree’ score 7 points. The score for each subscale is the sum of the points obtained on all the items; however, negatively worded items are scored inversely. The total scores range from 20 to 140 points. High scores indicate high levels of empathy. In the adaptation to Spanish, it has been shown to have high internal consistency and a reliability close to 90% (Cronbach’s Alpha: 0.82). The JSE is currently the most widely used instrument to measure this variable [19].
- Burnout. Burnout among healthcare professionals was measured using the Spanish version of the Maslach Burnout Inventory (MBI) [38]. It consists of 22 items in the form of statements about professionals’ feelings and attitudes towards their work and patients. Respondents rate the frequency with which they experience these feelings on a 7-point Likert scale ranging from 0 (never) to 6 (every day). The MBI includes 3 dimensions: emotional exhaustion (MBI-EE), who values the experience of being emotionally exhausted by labor demands, depersonalization (MBI-DP) examines the degree to which each person recognizes attitudes of emotional coldness and detachment, and personal accomplishment (MBI-PA) assesses feelings of self-efficacy and personal fulfillment at work. The score for each subscale is the sum of the points obtained on all the items; however, negatively worded items are scored inversely. High scores on MBI-EE and MBI-DP and low scores on MBI-PA indicate high levels of burnout [38]. In the adaptation to Spanish, this scale has a high internal consistency and a reliability close to 90% (Cronbach’s Alpha values: 0.90 for the emotional exhaustion; 0.79 for depersonalization, and 0.71 for personal accomplishment). Currently, the original MBI [39] is considered the most valued and most widely used instrument for measuring burnout in both national and international studies.
- Attitudes towards mentally ill patients were measured using the Spanish version of the Community Attitudes towards Mental Illness (CAMI) inventory [40]. The scale has been used in a variety of populations: nurses, psychiatrists, family members, as well as in the general population. It consists of 40 items rated on a 5-point Likert scale ranging from 1 (strongly agree) to 5 (strongly disagree) and is made up of 4 factors: authoritarianism (CAMI-A) designed to assess community attitudes toward the mentally ill, benevolence (CAMI-B) examines welcoming attitudes to patients, social restrictiveness (CAMI-SR) assesses the belief of danger to society of people with mental disorder, and community mental health ideology (CAMI-CMH) determines the beliefs related to the insertion into society of people with mental disorders. Each factor is divided into 10 statements addressing views on how to treat and care for individuals with a mental disorder. Five of the 10 items on each subscale are worded positively, while the other 5 are worded negatively. The score for each subscale is the sum of the points obtained on all the items; however, negatively worded items are scored inversely, i.e., when respondents give a score of 5 to an item expressed negatively, the score is considered to be 1. High scores on CAMI-B and CAMI-CMH and low scores on CAMI-A and CAMI-SR indicate high levels of attitudes towards mental health patients. In the adaptation to Spanish, this instrument has an internal consistency of 90% (Cronbach’s Alpha: 0.861).
- Other variables. The following sociodemographic data were collected: age, gender, years worked as a nurse, and years worked as a mental health nurse.

### 2.3. Statistical Analysis

Qualitative variables (gender) were described using absolute and relative frequencies. Quantitative variables (age, years working as a nurse, years working as a mental health nurse, JSE, MBI, and CAMI) were expressed as medians and interquartile ranges (IQR), as they showed significant deviations from the normal distribution according to the Kolmogorov–Smirnov test. The reliability of the JSE, MBI, and CAMI scales was assessed using Cronbach’s alpha. The correlation between the JSE, MBI, and CAMI scores and between these and sociodemographic variables was assessed using Spearman’s rank correlation coefficient (rho).

Comparisons of quantitative variables between two groups were performed using the Mann–Whitney U test and comparisons of quantitative variables between three or more groups were performed using the Kruskal–Wallis test. All tests were two-tailed, setting the statistical significance threshold at 0.05. The type 1 family-wise error rate was controlled at 0.05 by the Holm–Bonferroni correction [41]. Missing values were deleted in a pairwise fashion. There was only 0.03% of missing data and they were deleted in a pairwise fashion. All analyses were performed using IBM’s SPSS^©^ (version 25.0) software (IBM Corp., Armonk, NY, USA).

### 2.4. Ethical Considerations 

This research was approved by the Research Ethics Committee. The study strictly adheres to the general principles of bioethics, current legislation on health research, and Spanish Organic Law 3/2018, of the 5th of December, on Personal Data Protection and the Guarantee of Digital Rights. The privacy of the study participants was preserved at all times through the use of an anonymous database.

## 3. Results

The characteristics of the 750 study participants are shown in Table 1, Table 2, Table 3, Table 4 and Table 5. The majority of participants were female (62.5%) and had more than 20 years of professional experience as nurses (59.7%). Many were between 40 and 49 years of age (35.7%) and had also been working in mental health facilities for more than 20 years (47.6%) (Table 1).

The reliability of the scales used was good, meeting the criteria of being between the range of 0.7 and 0.9 [42], except for the MBI total score and the MBI-DP. In addition, Cronbach’s alpha analysis showed that the CAMI questionnaire had a low level of reliability (Cronbach’s alpha: 0.460). Detailed analysis of the scale items showed that most of the items were to be maintained, resulting in a decrease in the alpha value if removed, with the exception of items 9 and 29, whose removal increased the alpha value and were negatively correlated with the total. It was therefore decided to exclude them from the analysis (Cronbach’s alpha values: 0.779 for the JSE; 0.534 for MBI; 0.880 for MBI-EE; 0.524 for MBI-DP; 0.854 for MBI-PA; 0.817 for CAMI-A; 0.725 for CAMI-B; 0.850 for CAMI-SR, and 0.841 the CAMI-CMH). 

### 3.1. Empathy

Table 2 shows the results obtained for empathy. Significant differences (*p* < 0.05) were observed based on gender, age groups, years as an unspecialized nurse, and years as a mental health nurse. The level of empathy increased with an increase in these variables. A positive correlation (*p* < 0.05) can be observed between empathy and all the study variables, with the exception of the personal accomplishment dimension of burnout and the social restrictiveness and authoritarianism dimensions of attitudes towards mentally ill patients, where a negative correlation is observed (*p* < 0.05) (Table 3). These results mean that high empathy is associated with higher burnout since the higher the levels of empathy, the higher the levels of emotional exhaustion and depersonalization and the lower the levels of personal accomplishment which, as Maslach indicated, are the main prerequisites for causing burnout [38]. In addition, high empathy is associated with better attitudes towards mentally ill patients, since the higher the levels of empathy, the higher the levels of benevolence and community mental health ideology and the lower the levels of authoritarianism and social restrictiveness, as explained by the measurement instrument [40].

### 3.2. Burnout 

Table 4 shows the results obtained for burnout and its dimensions. Significant differences (*p* < 0.05) were observed in both total MBI and MBI dimensions between age groups, years as an unspecialized nurse, and years as a mental health nurse. No significant gender-based differences (*p* > 0.05) in the total MBI were observed, but they were observed in the MBI dimensions: men obtained higher values in the emotional exhaustion dimension and in the depersonalization dimension, while women obtained higher values in the personal accomplishment dimension. A positive correlation (*p* < 0.05) can be observed between the burnout total, emotional exhaustion and depersonalization dimensions of burnout and all the study variables, with the exception of the personal accomplishment dimension of burnout and the social restrictiveness and authoritarianism dimensions of attitudes towards mentally ill patients, with which a negative correlation is observed (*p* < 0.05). In addition, a negative correlation (*p* < 0.05) can be observed between the personal accomplishment dimension of burnout and all the study variables, with the exception of the dimensions of social restrictiveness and authoritarianism of attitudes towards patients with mental disorders, with which a positive correlation is observed (*p* < 0.05). All observed correlations were weak (>0.20) or moderate (>0.50), with the exception of the correlation between the total MBI and the dimensions of emotional exhaustion and depersonalization. The correlation between the emotional exhaustion dimension and personal accomplishment was also strong (>0.70) (Table 3).

### 3.3. Attitudes towards Mentally Ill Patients

Table 5 shows the results obtained for attitudes towards mentally ill patients and its dimensions. In the total CAMI there were no significant differences (*p* > 0.05) based on gender, years as an unspecialized nurse, and years as a mental health nurse, but there were significant differences between age groups (*p* < 0.05). Regarding the CAMI dimensions, significant differences (*p* < 0.05) were observed in relation to age groups, years as an unspecialized nurse, and years as a mental health nurse. Gender-based differences were observed only in the dimensions of authoritarianism and social restrictiveness, where women obtained higher values. A negative correlation (*p* < 0.05) can be observed between the authoritarianism dimension and all study variables, with the exceptions of the social restrictiveness dimension and the total score of attitudes towards patients with mental disorders, as well as the personal accomplishment dimension of burnout, where a positive correlation (*p* < 0.05) is observed. A positive correlation (*p* < 0.05) can be observed between the benevolence and community mental health ideology dimensions and all the study variables, with the exceptions of the social restrictiveness and authoritarianism dimensions of attitudes towards patients with mental disorders, as well as the personal accomplishment dimension of burnout, where a negative correlation is observed (*p* < 0.05). A negative correlation (*p* < 0.05) was identified between the social restrictiveness dimension and all study variables, with the exceptions of the authoritarianism dimension and the total score of attitudes towards mentally ill patients, as well as the personal accomplishment dimension of burnout, where a positive correlation (*p* < 0.05) is observed. All identified correlations were moderate (>0.50) or weak (>0.20), with the exception of the correlation between the dimensions of social restrictiveness and community mental health ideology (>0.70) (Table 3).

## 4. Discussion

Few studies have been conducted on the levels of empathy among mental health nurses [12,31]. Based on our results, participants’ empathy scores were relatively high, which is consistent with other nursing research findings [13] suggesting that psychiatric nurses exhibit the highest empathy scores [31]. With regard to gender, there is agreement in the literature that there are significant differences between men and women [12,13,14]. This is in line with our findings, which also revealed gender-based differences. Furthermore, empathy is directly related to the age of the professionals, the years worked as an unspecialized nurse, and the years worked as a mental health nurse, these results being in line with the international literature [13,31,43].

Burnout was found to increase with age and with the number of years worked as a nurse, especially as a mental health nurse. Although the scientific literature describes empathy as a protective factor against burnout [43,44], in our study we found that higher empathy scores indicate higher levels of burnout. A similar relation between empathy and burnout is reported by Bogiatzaki et al. [13] and Sturzu et al. [12], who attribute it to the intensity of the work involved in emotionally charged settings, such as mental health, compared to other healthcare settings. In these settings, the closeness of the nurse–patient relation and the management of difficult, challenging patient behaviors [45] result in mental health nurses having higher levels of burnout than in other specialties despite exhibiting high levels of empathy [12]. In our study, no significant differences in overall burnout were found between men and women; however, burnout dimensions were related to gender, as men showed higher scores than women on the dimensions of emotional exhaustion and depersonalization and lower scores on personal accomplishment. Bogiatzaki et al. [13] reported that gender is not a strong predictor of burnout, but it is true that scores differ between men and women, as shown in other studies [45]. Ferri et al. [15] state that depersonalization is related to distancing oneself from patients and may constitute a defense/protection mechanism. The results of a systematic review by Lopez-Lopez et al. [46] suggest that men are less well equipped than women to cope with work-related stress. Furthermore, Sturzu et al. [12] argue that violent incidents and the continuous demands of mental health patients can lead to emotional exhaustion. It is likely that men use the depersonalization mechanism to protect themselves from the stress caused by burnout [13] and that many of them experience emotional exhaustion due to the characteristics of patients with mental disorders [46]. The literature indicates that nurses maintain stability in their personal accomplishments while they are active in care work [47]. However, in our study we observed that personal accomplishment has a non-linear relationship with age, years as a nurse, and years in mental health. This is why there is an increase in personal accomplishment in middle-aged nurses, and after 5 years of working in mental health facilities, since according to the study by Alba-Martín [48] psychiatric services increase the motivational factor of the staff who work in them. This fact, together with the protective behaviors that can arise with patients suffering from a mental disorder [49] informs us of the significant changes in this dimension. However, it was also observed that personal accomplishment decreases in older nurses (40 years), from 16 years of experience as a nurse and, especially, from 11 years as a mental health specialist. This result is related to increased emotional exhaustion in this population [12]. Sturzu et al. [12] report that the continuous demands of mental health patients, as well as violent incidents, may lead to higher emotional exhaustion in older professionals and thus lower personal accomplishment.

There is little research on the attitudes mental health professionals hold towards patients with mental disorders [50]. In our study, the various dimensions of attitudes towards patients with mental disorders improve with age and work experience both in general care settings, and, especially and more significantly, in mental health settings, which is in line with a review by Sordi-Carrara et al. [50]. This improvement implies that the dimensions of benevolence and community mental health ideology increase and the dimensions of authoritarianism and social restrictiveness decrease. In addition, there are generally no differences between men and women, which is consistent with other studies [27,50]. However, women score higher than men in the dimensions of social restrictiveness and authoritarianism, which may be explained by their display of maternalistic attitudes in their relations with patients with mental disorders [49], given that gender plays an important social role according to Reupert et al. [51]. These attitudes render patients unable to make decisions for themselves and unable to take an active part in their own treatment, sometimes placing them in isolation for the sake of their health, with little contact with other individuals. It was also observed that a higher empathy score significantly improves overall attitudes towards mentally ill patients, as reported by Jung et al. [48]. Furthermore, if empathy is assessed by dimensions, higher levels of empathy are associated with higher levels of benevolence and community health and lower levels of authoritarianism and social restrictiveness among nurses. This phenomenon is linked to age, the number of years as a nurse and, specifically, the number of years as a mental health nurse, which is in consonance with Fradelos et al. [27] and Jung et al. [49].

## 5. Conclusions

This study suggests that high levels of empathy are associated with more positive attitudes towards patients with mental disorders, decreasing their associated stigma. In contrast, empathy did not act as a protective factor against burnout in the study sample. 

In addition, the high levels of burnout observed suggest that nurses working with mentally ill patients are highly likely to experience burnout despite their high levels of empathy.

### 5.1. Implications for Practice

Developing empathy skills through training programs would help to increase positive attitudes and decrease stigma towards patients with mental disorders, improving the quality of the care they receive. Futhermore, programs to reduce burnout among mental health professionals would thus be necessary to mitigate this problem and increase satisfaction in the work environment, especially in nurses who have been caring for these patients for longer.

Future studies should focus on observing how the study variables behave at other levels of the nursing career, such as nursing degree students and specialty residents being trained in mental health services. This would make it possible to design improvement and development programs specifically tailored to participants and to establish professional continuity in the development of empathy and the reduction of burnout.

### 5.2. Limitations

Firstly, the cross-sectional design of the study constitutes a limitation as it does not allow causal relations to be established. Secondly, the non-probability, non-discriminative, exponential snowball sampling method does not ensure the representativeness of the sample. Third, although this study used instruments adapted and validated in Spanish, and widely used to assess empathy and burnout, the JSE, the MBI, and the CAMI display a number of weaknesses relating to the complex definitions of the three variables they measure and their adaptation to cultures other than English-speaking ones [44,52,53]. Another limitation of our study is that the empathy, burnout and attitudes towards the mentally ill levels were based on questionnaire responses, and there is an obvious risk that the respondents may sometimes have answered what they thought was “expected”, rather than what they truly believed. Futhermore, the fact that the data were not normally distributed limited the type and complexity of the statistical analysis and hence the information provided by the data. Finally, the reliability of the scales was good, with the exception of the MBI total score. However, when analyzing the reliability by dimensions, we obtained better figures than those found by Yuguero et al. [44], closer to the reliability figures reported by the MBI questionnaire manual [54]. Like Yuguero et al. [44], we obtained low figures in the depersonalization dimension of burnout. Similar scores to the reliability of our studies can be observed in other studies as well, especially in non-English speaking populations [44,52].

## Figures and Tables

**Table 1:** Descriptive demographic and work-related data of the participants.

Variable	N = 750
Gender	
Male	281 (37.5%)
Female	469 (62.5%)
Age	
Male	47.0 (41.0–55.5)
Female	44.0 (36.0–54.0)
Age groups	
<30	25 (3.3%)
30–39	174 (23.2%)
40–49	268 (35.7%)
50–59	194 (25.9%)
>60	89 (11.9%)
Years as a nurse	
Male	25.0 (20.0–34.0)
Female	22.0 (14.0–31.0)
Groups of years as a nurse	
<5	22 (2.9%)
5–10	78 (10.4%)
11–15	75 (10.0%)
16–20	127 (16.9%)
>20	448 (59.7%)
Years in mental health	
Male	22.0 (16.0–30.0)
Female	19.0 (10.0–28.0)
Groups of years in mental health	
<5	67 (8.9%)
5–10	95 (12.7%)
11–15	86 (11.5%)
16–20	145 (19.3%)
>20	357 (47.6%)

Quantitative variables are expressed as medians (IQRs). Qualitative variables are expressed as frequencies (%).

**Table 2:** Association between empathy and sociodemographic variables.

Variable	Total Empathy Median (IQR)	*p*-Value
Gender	130.0 (128.0–132.0)	0.031
Male	130.0 (129.0–132.0)	
Female	130.0 (128.0–132.0)	
Age groups		0.000
<30	124.0 (121.0–129.5)	
30–39	129.0 (126.0–130.0)	
40–49	130.0 (129.0–131.0)	
50–59	131.0 (128.0–132.0)	
>60	132.0 (130.5–133.0)	
Years as a nurse		0.000
<5	126.0 (121.0–129.25)	
5–10	127.0 (124.0–130.0)	
11–15	129.0 (127.0–130.0)	
16–20	130.0 (128.0–131.0)	
>20	131.0 (129.0–132.0)	
Years in mental health		0.000
<5	126.0 (121.0–129.0)	
5–10	129.0 (126.0–130.0)	
11–15	130.0 (128.0–131.0)	
16–20	130.0 (128.0–131.0)	
>20	131.0 (129.0–132.0)	

**Table 3:** Spearman’s rank correlation matrix between study variables.

VARIABLES	Empathy (JSE)	Total Burnout (MBI)	Emotional Exhaustion (MBI-EE)	Depersonalization (MBI-DP)	Personal Accomplishment (MBI-PA)	Total Community Attitudes towards Mental Illness (CAMI)	Authoritaria-nism (CAMI-A)	Benevolence (CAMI-B)	Social Restrictiveness (CAMI-SR)	Community Mental Health Ideology (CAMI-CMH)	Age	Years as a Nurse	Years in Mental Health
JSE	1												
Total MBI	0.247 **	1											
MBI-EE	0.204 **	0.817 **	1										
MBI-DP	0.179 **	0.717 **	0.656 **	1									
MBI-PA	−0.072*	−0.299 **	−0.732 **	−0536 **	1								
Total CAMI	0.072*	0.279 *	0.251 **	0.171 **	−0.087 *	1							
CAMI-A	−0.419 **	−0.170 **	−0.226 **	−0.250 **	0.252 **	0.214 **	1						
CAMI-B	0.292 **	0.200 **	0.140 **	0.152 **	−0.024	0.398 **	−0.323 **	1					
CAMI-SR	−0.396 **	−0.170 **	−0.228 **	−0.207 **	0.239 **	0.121 **	0.693 **	−0.397 **	1				
CAMI-CMH	0.424 **	0.315 **	0.377 **	0.292 **	−0.308 **	0.416 **	−0.602 **	0.473 **	−0.733 **	1			
Age	0.360 **	0.405 **	0.581 **	0.468 **	−0.568 **	−0.006	−0.549 **	0.180 **	−0.522 **	0.525 **	1		
Years as a nurse	0.367 **	0.378 **	0.555 **	0.432 **	−0.543 **	−0.009	−0.560 **	0.187 **	−0.536 **	0.533 **	0.973 **	1	
Years in mental health	0.392 **	0.409 **	0.566 **	0.451 **	−0.527 **	−0.008	−0.569 **	0.181 **	−0.541 **	0.538 **	0.942 **	0.962 **	1

* indicates a significant correlation, *p* < 0.05. ** indicates a significant correlation, *p* < 0.01.

**Table 4:** Association between burnout and sociodemographic variables.

Variable	Total Burnout (MBI)	Emotional Exhaustion (MBI-EE)	Depersonalization (MBI-DP)	Personal Accomplishment (MBI-PA)
Gender	55.0 (51.0–59.0)	11.0 (6.0–15.0)	3.0 (2.0–4.0)	41.0 (38.0–44.0)
Male	55.0 (52.0–60.0)	12.0 (7.0–16.0)	3.0 (2.0–4.5)	41.0 (38.0–43.0)
Female	55.0 (51.0–58.0)	10.0 (5.0–15.0)	2.0 (2.0–4.0)	42.0 (38.0–45.0)
*p*-value	0.106	0.033	0.003	0.015
Age groups				
<30	52.0 (46.0–66.0)	8.0 (2.0–22.5)	2.0 (0.0–5.5)	39.0 (35.5–45.0)
30–39	52.0 (49.0–55.0)	4.0 (2.0–9.0)	2.0 (1.0–3.0)	46.0 (43.0–47.5)
40–49	54.0 (50.0–57.0)	9.0 (6.0–13.0)	2.0 (2.0–3.0)	42.0 (41.0–44.0)
50–59	57.0 (53.0–61.0)	14.0 (11.0–16.0)	4.0 (2.0–5.0)	40.0 (37.0–41.0)
>60	59.0 (56.0–64.0)	18.0 (15.0–21.0)	5.0 (3.5–7.0)	36.0 (34.0–39.0)
*p*-value	0.000	0.000	0.000	0.000
Years as a nurse				
<5	50.0 (46.0–59.25)	7.0 (0.0–16.0)	2.0 (0.0–4.25)	43.5 (36.75–47.0)
5–10	52.0 (48.0–56.25)	4.0 (2.0–11.25)	2.0 (1.0–3.0)	46.0 (41.0–47.0)
11–15	52.0 (49.0–56.0)	5.0 (2.0–8.0)	2.0 (1.0–3.0)	46.0 (42.0–48.0)
16–20	54.0 (49.0–57.25)	8.0 (4.0–11.0)	2.0 (1.0–3.0)	43.5 (41.0–46.0)
>20	56.0 (53.0–60.0)	13.0 (10.0–16.0)	3.0 (2.0–5.0)	40.0 (37.0–42.0)
*p*-value	0.000	0.000	0.000	0.000
Years in mental health				
<5	51.0 (47.0–56.0)	4.0 (0.0–16.0)	2.0 (0.0–3.0)	45.0 (37.0–47.0)
5–10	52.0 (50.0–56.0)	4.0 (2.0–8.0)	2.0 (1.0–3.0)	47.0 (42.0–48.0)
11–15	53.0 (48.0–57.0)	7.0 (4.0–10.25)	2.0 (1.0–3.0)	44.0 (41.0–46.0)
16–20	54.0 (50.0–57.0)	9.0 (6.0–12.0)	2.0 (2.0–3.0)	42.0 (41.0–44.0)
>20	57.0 (54.0–61.0)	14.0 (11.0–17.0)	3.0 (3.0–5.0)	40.0 (37.0–42.0)
*p*-value	0.000	0.000	0.000	0.000

**Table 5:** Association between attitudes towards mental health patients and sociodemographic variables.

Variable	Total Community Attitudes towards Mental Illness (CAMI)	Authoritarianism (CAMI-A)	Benevolence (CAMI-B)	Social Restrictiveness (CAMI-SR)	Community Mental Health Ideology (CAMI-CMH)
Gender	114.0 (113.0–115.0)	9.0 (8.0–9.0)	46.0 (45.2–46.0)	12.0 (10.0–13.0)	47.0 (45.0–49.0)
Male	114.0 (113.0–115.0)	9.0 (8.0–9.0)	46.0 (46.0–46.0)	12.0 (10.0–13.0)	47.0 (45.0–50.0)
Female	114.0 (113.0–115.0)	9.0 (8.0–10.0)	46.0 (45.0–46.0)	13.0 (11.0–14.0)	47.0 (45.0–49.0)
*p*-value	0.136	0.000	0.127	0.003	0.056
Age groups					
<30	114.0 (113.0–116.5)	12.0 (10.0–15.5)	45.0 (44.0–46.0)	16.0 (13.0–18.0)	44.0 (38.0–47.0)
30–39	114.0 (112.7–116.0)	9.0 (9.0–11.0)	46.0 (45.0–46.0)	13.0 (12.0–14.25)	45.0 (44.0–47.0)
40–49	114.0 (112.0–115.0)	9.0 (8.0–9.0)	46.0 (46.0–46.0)	13.0 (11.0–13.0)	46.0 (45.0–48.0)
50–59	114.0 (113.0–115.0	8.0 (8.0–9.0)	46.0 (46.0–46.0)	11.0 (10.0–13.0)	49.0 (46.0–50.0)
>60	114.0 (114.0–114.0)	8.0 (8.0–8.0)	46.0 (46.0–46.0)	10.0 (10.0–10.0)	50.0 (50.0–50.0)
*p*-value	0.018	0.000	0.000	0.000	0.000
Years as a nurse					
<5	114.0 (113.0–15.25)	10.0 (9.0–13.5)	45.5 (44.0–46.0)	15.0 (12.0–17.25)	44.0 (39.75–46.25)
5–10	114.5 (112.0–117.25	10.0 (9.0–12.0)	46.0 (45.0–46.0)	14.0 (12.0–15.0)	45.0 (42.75–47.25)
11–15	114.0 (113.0–115.0)	9.0 (9.0–10.0)	46.0 (45.0–46.0)	13.0 (12.0–14.0)	46.0 (44.0–47.0)
16–20	114.0 (112.0–115.0)	9.0 (9.0–9.0)	46.0 (45.0–46.0)	13.0 (12.0–14.0)	46.0 (44.0–48.0)
>20	114.0 (113.0–114.0)	8.0 (8.0–9.0)	46.0 (46.0–46.0)	11.0 (10.0–13.0)	48.0 (46.0–50.0)
*p*-value	0.208	0.000	0.000	0.000	0.000
Years in mental health					
<5	114.0 (112.0–117.0)	10.0 (9.0–12.0)	46.0 (44.0–46.0)	14.0 (12.0–16.0)	45.0 (41.0–47.0)
5–10	114.0 (112.0–115.0)	9.0 (9.0–10.0)	46.0 (45.0–46.0)	13.0 (13.0–14.0)	45.0 (43.0–47.0)
11–15	114.0 (112.0–115.0)	9.0 (9.0–9.0)	46.0 (45.0–46.0)	13.0 (12.0–14.0)	45.5 (44.0–48.0)
16–20	114.0 (112.0–115.0)	9.0 (9.0–9.0)	46.0 (45.0–46.0)	13.0 (11.0–13.0)	46.0 (45.0–48.0)
>20	114.0 (113.0–114.0)	8.0 (8.0–9.0)	46.0 (46.0–46.0)	11.0 (10.0–13.0)	49.0 (46.0–50.0)
*p*-value	0.220	0.000	0.000	0.000	0.000

Median (IQR), Total CAMI = community attitudes towards mental illness; CAMI-A = authoritarianism; CAMI-B = benevolence; CAMI-SR = social restrictiveness; CAMI-CMH = community mental health ideology.
